# Pituitary Adenlylate Cyclase Activating Peptide Protects Adult Neural Stem Cells from a Hypoglycaemic *milieu*

**DOI:** 10.1371/journal.pone.0156867

**Published:** 2016-06-15

**Authors:** Shiva Mansouri, Grazyna Lietzau, Mathias Lundberg, David Nathanson, Thomas Nyström, Cesare Patrone

**Affiliations:** Department of Clinical Science and Education, Södersjukhuset, Internal Medicine, Karolinska Institutet, Stockholm, Sweden; University of Rouen, FRANCE

## Abstract

Hypoglycaemia is a common side-effect of glucose-lowering therapies for type-2 diabetic patients, which may cause cognitive/neurological impairment. Although the effects of hypoglycaemia in the brain have been extensively studied in neurons, how hypoglycaemia impacts the viability of adult neural stem cells (NSCs) has been poorly investigated. In addition, the cellular and molecular mechanisms of how hypoglycaemia regulates NSCs survival have not been characterized. Recent work others and us have shown that the pituitary adenylate cyclase-activating polypeptide (PACAP) and the glucagon-like peptide-1 receptor (GLP-1R) agonist Exendin-4 stimulate NSCs survival against glucolipoapoptosis. The aim of this study was to establish an *in vitro* system where to study the effects of hypoglycaemia on NSC survival. Furthermore, we determine the potential role of PACAP and Exendin-4 in counteracting the effect of hypoglycaemia. A hypoglycaemic *in vitro milieu* was mimicked by exposing subventricular zone-derived NSC to low levels of glucose. Moreover, we studied the potential involvement of apoptosis and endoplasmic reticulum stress by quantifying protein levels of Bcl-2, cleaved caspase-3 and mRNA levels of CHOP. We show that PACAP *via* PAC-1 receptor and PKA activation counteracts impaired NSC viability induced by hypoglycaemia. The protective effect induced by PACAP correlated with endoplasmic reticulum stress, Exendin-4 was ineffective. The results show that hypoglycaemia decreases NSC viability and that this effect can be substantially counteracted by PACAP *via* PAC-1 receptor activation. The data supports a potential therapeutic role of PAC-1 receptor agonists for the treatment of neurological complications, based on neurogenesis impairment by hypoglycaemia.

## Introduction

Worldwide the number of prevalent cases of type 2 diabetes (T2D) has increased from 150 million in 1980 to 347 million in 2011 [1]. T2D increases the risk of developing cardiovascular diseases (CVD) and central nervous system (CNS) complications such as stroke, dementia and Alzheimer’s disease (AD) [2, 3]. Although clinical data about the efficacy of glucose lowering treatments are unclear when it comes to reduce the incidence of CVD and CNS complications in T2D [4], applying intensive glucose control therapy represents today one, among others strategies, for avoiding such a complications [5]. However, glucose lowering medications in T2D can upset the homeostatic balance between blood glucose and insulin levels, resulting in the development of hypoglycaemia [6]. The detrimental effects mediated by hypoglycaemia can lead to oxidative stress causing cardiac arrhythmias and contribute to sudden cardiac arrest [7]. In particular, this has been mainly reported by using combinations of glucose-lowering medications such as sulfonylureas and insulin [8] and may become a health concern [9]. In recent years evidence has grown showing that episodes of hypoglycaemia can also affect the brain by increasing the risk of developing dementia and AD [9–12]. Moreover, hypoglycaemia can also increase ischemic cerebral damage [13, 14].

The brain is highly sensitive to hypoglycaemia since it largely relies on glucose for metabolism and this may result in neuronal loss due to impaired fuel supply. Studies have shown that hypoglycaemia can cause significant neuronal cell loss in hippocampal and cortical neurons both *in vitro* [15–17] and *in vivo* [18, 19]. As a consequence, impairment of a variety of neuropsychological functions in diabetic patients, including memory has been reported [20–25]. The mechanisms leading to hypoglycaemia-mediated neuronal cell death have been recently investigated. Several factors have been suggested to play a role in the detrimental effects mediated by hypoglycaemia in the brain such as excitotoxicity [26, 27], nitric oxide production, zinc release from nerve terminals, the activation of poly-(ADP ribose) polymerase-1 (PARP-1) and inhibition of autophagy [28–31]. Moreover, studies have shown the involvement of caspase-dependent and independent mitochondrial apoptosis, observed in the hippocampus of hypoglycaemic rats [32, 33]. In addition, a recent study showed the involvement of endoplasmic reticulum (ER) stress in triggering apoptotic neuronal death by glucose deprivation in hippocampal cultured neurons [17].

Today, we recognize that in the adult mammalian brain, a proliferating population of neural stem cells (NSCs) are generated throughout adulthood life *via* a mechanism known as adult neurogenesis [34]. Neurogenesis occurs in three primary areas in the nervous system: the subgranular zone (SGZ), which supplies new granule cells to the dentate gyrus (DG) of the hippocampus; the subventricular zone (SVZ), which supplies new interneurons to the olfactory bulb in rodent [35] and also to the striatum in the human brain [36], and recently neurogenesis has been also described in the hypothalamic region [37]. The precise role of adult neurogenesis under physiological and pathological conditions is still largely unknown [38], although several data suggest the importance of this process in the regulation of neurologic [39] and metabolic [40] functions.

How neurogenesis is impacted by hypoglycaemic conditions has been partially investigated in the hippocampus. Suh *et al*. showed that during the first 2 weeks after hypoglycaemia induced in rats, a transient increase of progenitor cell proliferation in the SGZ and neurogenesis in the hippocampal granule cell layer (GCL) was observed. However, after 4 weeks a significant loss of progenitor cells in the SGZ was seen [18]. In addition, work by Estrada *et al*. showed that an experimental model of transient hypoglycemia increased astrogliosis as well as neurogenesis in the rat hippocampus, suggesting that the brain undergoes a repairing process after injury [41]. Finally, an experimental model of repetitive hypoglycaemia failed to show neurogenesis impairment in the hippocampal DG, yet showing selective impairment of synaptic plasticity in the absence of cell death [42].

The aim of this study was to determine the direct effect of hypoglycaemia on primary NSCs isolated from the SVZ of the adult mouse. Furthermore, we aimed to identify some of the mechanisms at the basis of the hypoglycaemic effects on NSCs. Others and we have previously shown that the pituitary adenylate cyclase-activating polypeptide (PACAP) regulates NSCs viability *in vitro* and *in vivo* [43–47]. Moreover, we have shown that the glucagon-like peptide-1 (GLP-1) receptor agonist Exendin-4 (Ex-4) enhances neural progenitor cells (NPCs) survival in a diabetic *milieu in vitro* [48] and *in vivo* [49–51]. Furthermore while PACAP has been shown to contribute to the glucagon response to insulin-induced hypoglycaemia in mice [52] administration of Ex-4 did not produce hypoglycemic effects in T2D patients [53]. Thus, we also aimed to determine the potential role of PACAP and Ex-4 in regulating NSCs viability under hypoglycaemic conditions.

## Materials and Methods

### NSCs isolation and cell cultures

The SVZ of the lateral brain ventricles of adult male mice 6 weeks of age (five C57 BL6/SCA mice in each experiment) was micro-dissected by using a micro-dissector scissor and enzymatically dissociated in 0.5 mg/ml trypsin, 0.8 mg/ml hyaluronidase and 80 U/ml deoxyribonuclease I (Sigma-Aldrich, St Louis, MO) in DMEM/F12 containing B27 supplement, 4.5 mg/ml glucose, 100 U/mL penicillin, 100 μg/ml streptomycin sulfate and 12.5 mM HEPES buffer solution (Invitrogen, Stockholm, Sweden). The enzymatic digestion was carried out at 37°C for 20 min. After a gentle trituration with a pipette and mixing, cells were passed through a 70 μm strainer (BD Biosciences, Stockholm, Sweden) and pelleted at 1,000 rpm for 12 min. The centrifugation step was repeated once more after removing the supernatant by adding fresh cold DMEM/F12. The supernatant was then removed, and cells were re-suspended in DMEM/F12 supplemented with B27 and 18 ng/ml human epidermal growth factor (EGF) (R&D systems, Oxon, U.K.). Cells were plated in a 10 cm Petri dish and incubated at 37°C for 7 days in order for neurospheres (NS) to be developed. After 7 days, the NS were collected and centrifuged at 1,000 rpm for 10 min. NS were re-suspended in 0.5% trypsin/EDTA (Invitrogen, Stockholm, Sweden), by incubating at 37°C for 2 min and triturated gently to aid dissociation. After a further 2 min incubation at 37°C, the cell preparation was diluted 1/20 in DMEM/F12 at 37°C. Cells were then pelleted at 1,000 rpm for 10 min and re-suspended in fresh DMEM/F12 containing 18 ng/ml EGF and 16 ng/ml human basic fibroblast growth factor (bFGF) (R&D systems, Oxon, U.K.) before plating. NS were passed every 5 days for 4 weeks and all experiments were performed between passage 2 and 8.

### Hypoglycaemic medium

To mimic a hyperglycaemic *milieu in vitro*, DMEM medium without D-glucose was used. In the experiments, cells were maintained in 0.01 ng/ml of EGF to avoid high proliferative conditions. To obtain the desired glucose concentration, the NSC medium was supplemented with different concentration of glucose ranging from 20 mM (control) to 0 mM (concentrations shown in the results section).

### ATP assay

Previous reports have indicated that intracellular ATP levels correlate to cell numbers [54]. To measure NSC viability, NSCs were plated as single cells (see above) into 96-well plates (Corning B.V. Life Sciences, Amsterdam, Netherlands) at the final concentration of 30,000 cells/well with DMEM medium without D-glucose supplemented with B27. PACAP 38 (Phoenix Pharmaceuticals, Burlingame, CA, USA), Maxadilan-4 (Max4; PAC-1 specific agonist; graciously provided by R.G. Titus), Exendin-4 (Ex-4; Sigma Aldrich, St Louis, MO, USA) were added to NSC for 15 min before or immediately after exposing the cells to hyperglycaemic medium at the concentrations shown in the results section. The treatments were maintained for 24 hours. H89 (PKA-inhibitor) 1μM, Gö-6976 (PKC-inhibitor) 1μM (Sigma-Aldrich) and PACAP 6–38 (a specific PAC-1/VPAC2 antagonist) 1μM was administered to the cells 10 min before PACAP 38 and 2.5mM glucose. After 24 hours of incubation at 37°C (5% CO_2_, 98% humidity), intracellular ATP levels were measured using the Cellular ATP Kit HTS according to the manufacturer’s instructions (BioThema, Stockholm, Sweden). In these experiments, the effect of each treatment at a certain concentration was between 4–8 samples in 6–7 different sets of experiments.

### NSC counting

To measure NSC number, NSCs were plated as single cells into 96-well plates at the final concentration of 30,000 cells/well with DMEM medium without D-glucose supplemented with B27 (see above). PACAP 38 was added to NSC for 15 min before exposing the cells to hyperglycaemic medium at the concentrations shown in the results section. The treatments were maintained for 24 hours. After 24 hours of incubation cell counting (and small spheres formed along the 24 hours incubation time) was performed manually in two fields of each well.

### Western blot

NSCs were plated as single cells and expanded in a 10 cm Petri dish with EGF/bFGF (see under cell cultures) for 3–4 days. When NS were formed, the different treatments were added for 24 hours. After exposure, cells were washed with PBS and lyseded in a buffer containing 150mM NaCl, 20mM Tris, 0.1% SDS, 1% Triton X-100, 0.25% Na-deoxycholate, 1mM Na3VO4, 50mM NaF, 2mM EDTA, and Protease inhibitory cocktail (Sigma–Aldrich) on ice for 30 min. Samples were clarified by centrifugation. The supernatants were transferred to new tubes and the total protein concentration was determined by Lowry protein assay (Bio-Rad Laboratories, Stockholm, Sweden). Samples were then mixed with reducing SDS-PAGE sample buffer and boiled for 5 min before performing SDS-PAGE. After electrophoresis, proteins were transferred onto polyvinylidene fluoride (PVDF) membranes (Bio-Rad Laboratories). Immunoblot analyses were performed with antibodies against the cleaved form of caspase-3 (1:1,000, polyclonal) (Cell Signaling Technology, Danvers, MA, USA) and Bcl-2 1:800 monoclonal rabbit,) (Abcam, Cambridge, MA, USA). Immuno-reactive bands were developed using ECL (GE Healthcare, Stockholm, Sweden), imaged with a GelDoc system and quantified with Quantity One software (Bio-Rad Laboratories). After imaging, to verify equal protein loading, the PDVF membranes were normalized versus β-actin protein levels (1:800, polyclonal) (Santa Cruz Biotechnology, Inc. Germany). In these experiments, the effect of each treatment at a certain concentration was determined in single samples in 5–8 different set of experiments.

### Quantitative RT-PCR

To quantify the ER stress-inducible transcription factor CHOP mRNA levels the total RNA was extracted using Aurum total RNA-mini kit (Bio-Rad Laboratories, Stockholm, Sweden) and the RNA was treated with DNase I (Bio-Rad Laboratories, Stockholm, Sweden) to eliminate possible DNA contamination, according to the manufacturer’s protocol. Total mRNA was reversed transcribed into cDNA by using an iScript^™^cDNA Synthesis Kit (Bio-Rad Laboratories, Stockholm, Sweden). The expression levels of mRNAs were measured by SYBR green based quantitative RT-PCR (iQ^™^ SYBR^®^ Green Supermix; Fermentas, St. Leon-Rot, Germany) using mouse-specific primer pairs for CHOP (forward:5'-GAAAGCAGAACCTGGTCCAC-3, reverse: 5'-GACCTCCTGCAGATCCTCAT-3′) (Invitrogen, Stockholm, Sweden). β-actin was used as an internal standard.

### Statistical analysis

Differences between groups were tested with one-way ANOVA followed by post hoc Fisher LSD test or Kruskal-Wallis followed by Dunn’s test if data was not normally distributed. All statistical analyses were performed using Sigma Plot software v. 11. Data are presented as mean ±SEM. *P* < 0.05 was considered statistically significant.

All experiments were conducted according to the regional ethics committee for animal experimentation conforming to the "Guide for the Care and Use of Laboratory Animals" published by U.S. National Institutes of Health (NIH publication # 85–23, revised 1985). The C57 BL6/J mice were imported from Nova-SCB, Stockholm, Sweden. The regional ethical committee (Stockholm Södra djurförsöksetiska nämnd) has approved all animals studies presented in the manuscript. For animal experimentation (application S72-12). Animals were sacrificed in CO_2_ chamber and by cervical dislocation.

## Results

### A hypoglycaemic *milieu* impairs NSC viability

**Fig 1:**
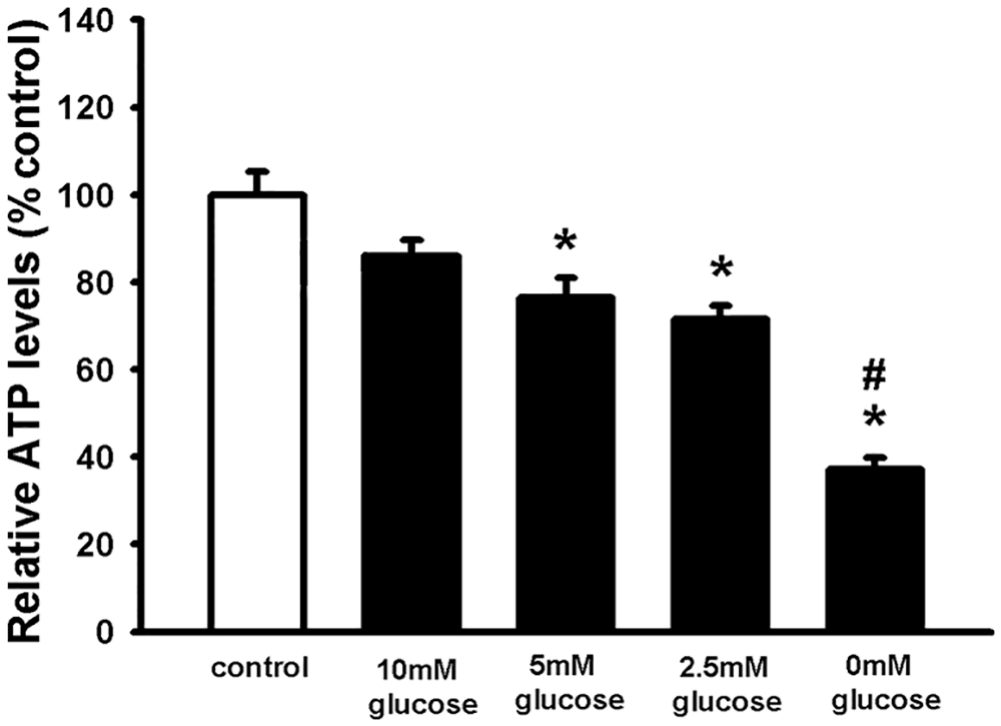
A hypoglycaemic *milieu* decreases NCS viability in a dose-dependent manner. NSCs were plated as single cells and treated with 20 mM (control) 10, 5, 2.5, 0 mM glucose. To measure cell viability, intracellular ATP levels were measured after 24 hours. Values are shown as mean ±SEM (n = 14–25). Kruskal-Wallis followed by Dunn’s test was used. Differences were considered significant at P <0.05. * denotes P <0.05 compared with control, # denotes P<0.05 compared to 2.5mM glucose.

To study the effect of a hypoglycaemic *milieu* on NSC viability, hypoglycaemic conditions were mimicked by adding different concentrations of glucose [20 mM (control conditions) 10 mM, 5 mM, 2.5 mM, and 0 mM] to NSCs isolated from the SVZ of the adult mouse. NSC viability was assessed after 24 hours by measuring intracellular ATP levels. The results showed that low glucose levels from 5 mM impair NSC viability in a dose-dependent manner (Fig 1). Severe or profound hypoglycaemia is usually referred to glucose levels of 2.5 mM or less [55, 56]. Therefore, this glucose concentration was used in the following experiments.

### Decreased NSC survival by hypoglycaemia correlates with a modest increase in apoptosis

**Fig 2:**
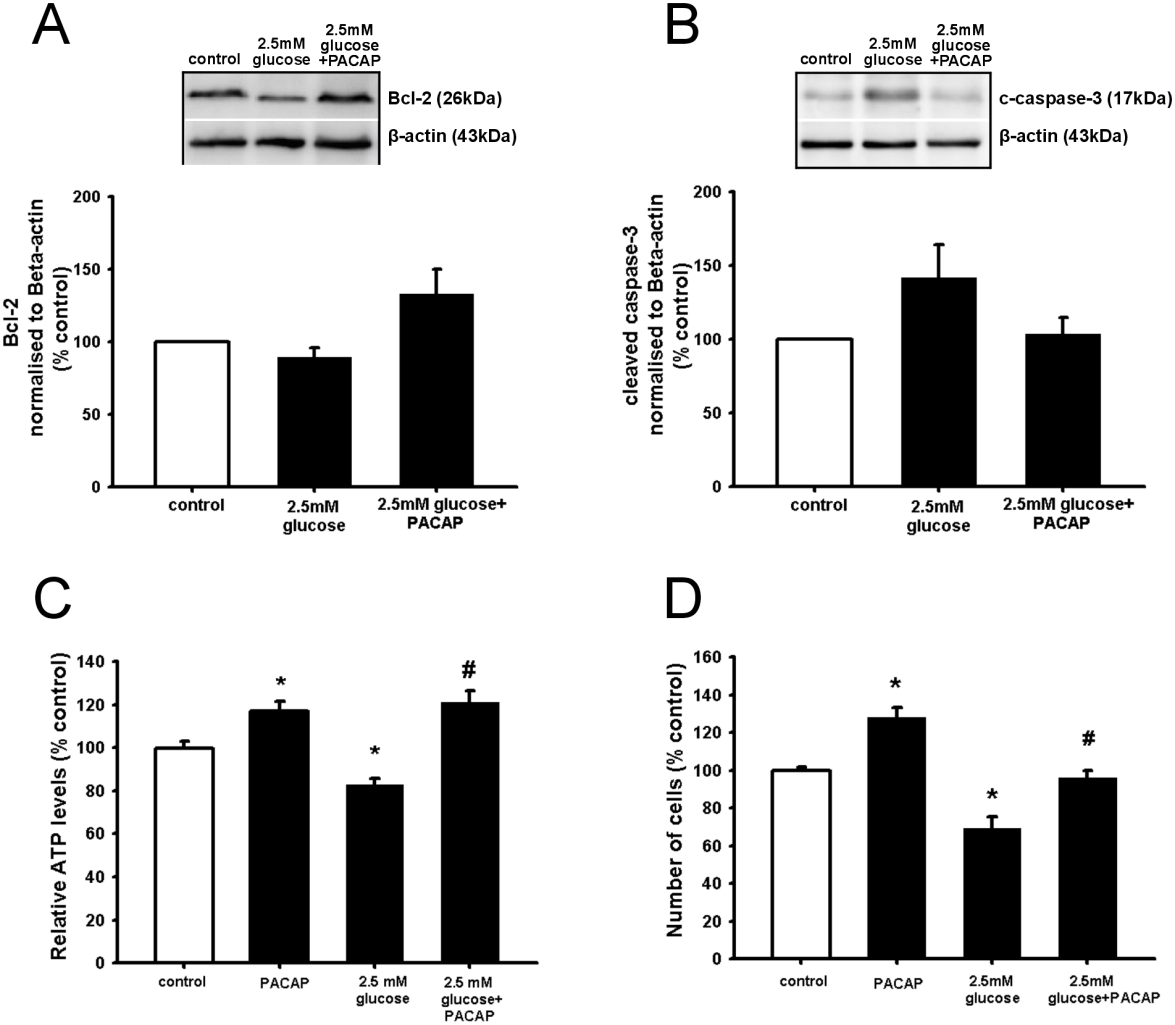
PACAP increases NSC viability in response to hypoglycaemic *milieu* in correlation with a downward trend in apoptosis. NSCs were plated as single cells. Prior to 2.5mM glucose addition cells were incubated with PACAP (100 nM) for 10 min. After 24 hours incubation cells were harvested for Western blot experiments **(A, B)** or intracellular ATP determination **(C)** or NSC manual counting **(D)**. To obtain quantitative measurements Bcl-2 protein levels and cleaved caspase 3 were normalized against β-actin. Data are shown as mean ±SEM (A, n = 6–7; B, n = 5–8, C, n = 30–50; D, n = 3–4). Kruskal-Wallis followed by Dunn’s test or Fisher LSD test was used. Differences were considered significant at P<0.05. * denotes P <0.05 compared with control, # denotes P<0.05 compared to 2.5mM glucose.

To study the potential involvement of apoptosis in the decreased NSC viability induced by hypoglycaemia, we assessed the levels of the pro-apoptotic cleaved form of caspase-3 and of the anti-apoptotic protein Bcl-2 after 24 hours of incubation in 2.5 mM glucose, by Western blotting analysis. The results showed that low glucose (2.5mM) induced a trend toward the decrease of Bcl-2 protein levels (Fig 2A) while there was a trend toward the increase in the protein levels of the cleaved form of caspase-3 (Fig 2B), (S1 Fig).

### PACAP counteracts impaired NSCs viability by hypoglycaemia in correlation with decreased apoptosis and ER stress

Previous results from our group have shown that PACAP significantly counteracts glucolipotoxicity in correlation with decreased apoptosis [57]. Therefore, to determine whether PACAP had similar effect in a hypoglycaemic *milieu*, we pre-treated NSCs with PACAP (100 nM) before exposing the cells to 2.5 mM glucose. NSCs viability was determined 24 hours after. As expected, the results in Fig 2C showed that PACAP increases NSC viability under normal conditions (previously been reported [43, 47]. Moreover, PACAP was able to counteract entirely the negative hypoglycaemic effect on NSCs (Fig 2C). The results were also confirmed by manual cell counting (Fig 2D). In another set of experiments our data shows that PACAP-mediated NSC protection lasted until 48 hours in culture following PACAP pre-treatment (data not shown). In addition PACAP was also able to induce cell protection when the peptide was added a few minutes after the hypoglycemic condition (data not shown). Next we addressed the role of PACAP in counteracting apoptosis in a hypoglycaemic *milieu* by assessing Bcl-2 and cleaved-form of caspase-3 by Western blotting analysis. The results in Fig 2A and 2B showed that PACAP was able to induce non-statistically significant trends toward an increase of the protein levels of Bcl-2 and toward a decrease of cleaved caspase-3 protein expression.

**Fig 3:**
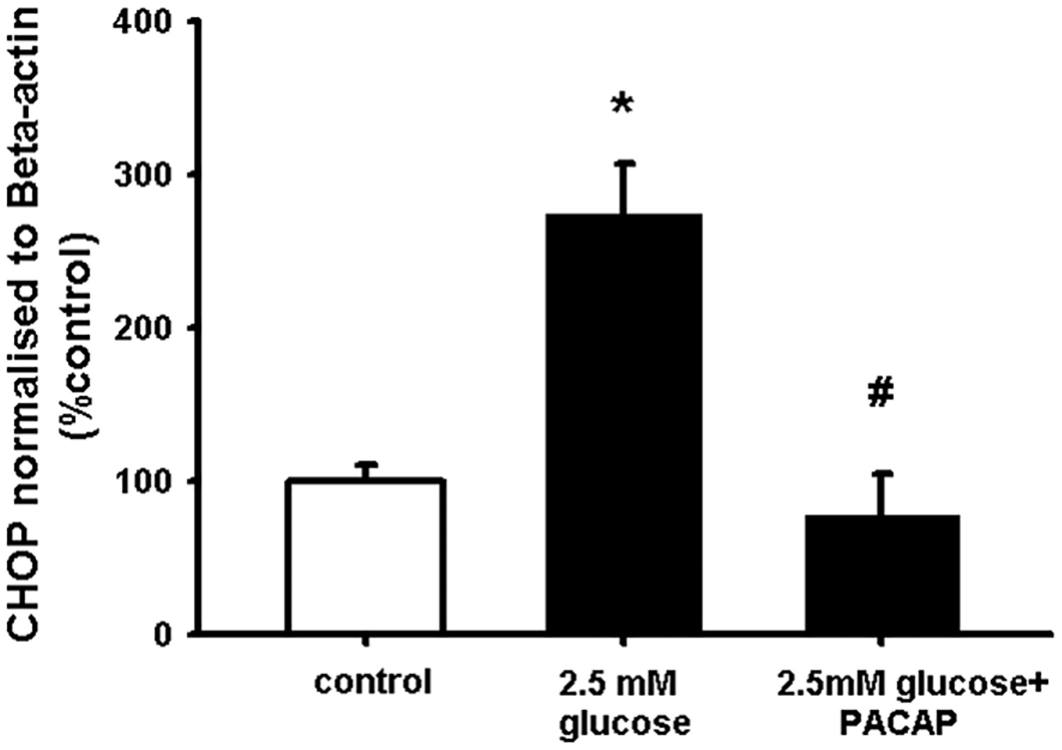
PACAP counteracts hypoglycaemia-induced ER stress in NSCs. NSCs were plated as single cells. Prior to 2.5mM glucose addition cells were incubated with PACAP (100 nM) for 10 min. After 24 hours incubation, cells were harvested for mRNA extraction and quantitative RT-PCR experiments. Data are shown as mean ±SEM (n = 3–4). Kruskal-Wallis followed by Dunn’s test was used. Differences were considered significant at P<0.05. * denotes P <0.05 compared with control, # denotes P<0.05 compared to 2.5mM glucose.

ER stress plays an important role in the development and pathology of diabetes and neurodegenerative diseases [58, 59]. To study whether hypoglycaemia induces ER stress and whether PACAP can counteract this effect, NSCs were pre-treated with PACAP before being exposed to low glucose. After 24 hours, quantitative RT-PCR for the mRNA levels of CHOP was performed [60]. The results in Fig 3 show that the ER stress marker CHOP was significantly up-regulated in the presence of hypoglycaemia in comparison to control. The results also show that PACAP was able to abolish the effect of hypoglycaemia on increased CHOP mRNA levels (Fig 3). These data indicate that the activation of PACAP decreases ER stress under hypoglycaemic conditions.

### The PACAPs neuroprotective effect is mediated *via* PAC-1 activation through the PKA-dependent pathway

**Fig 4:**
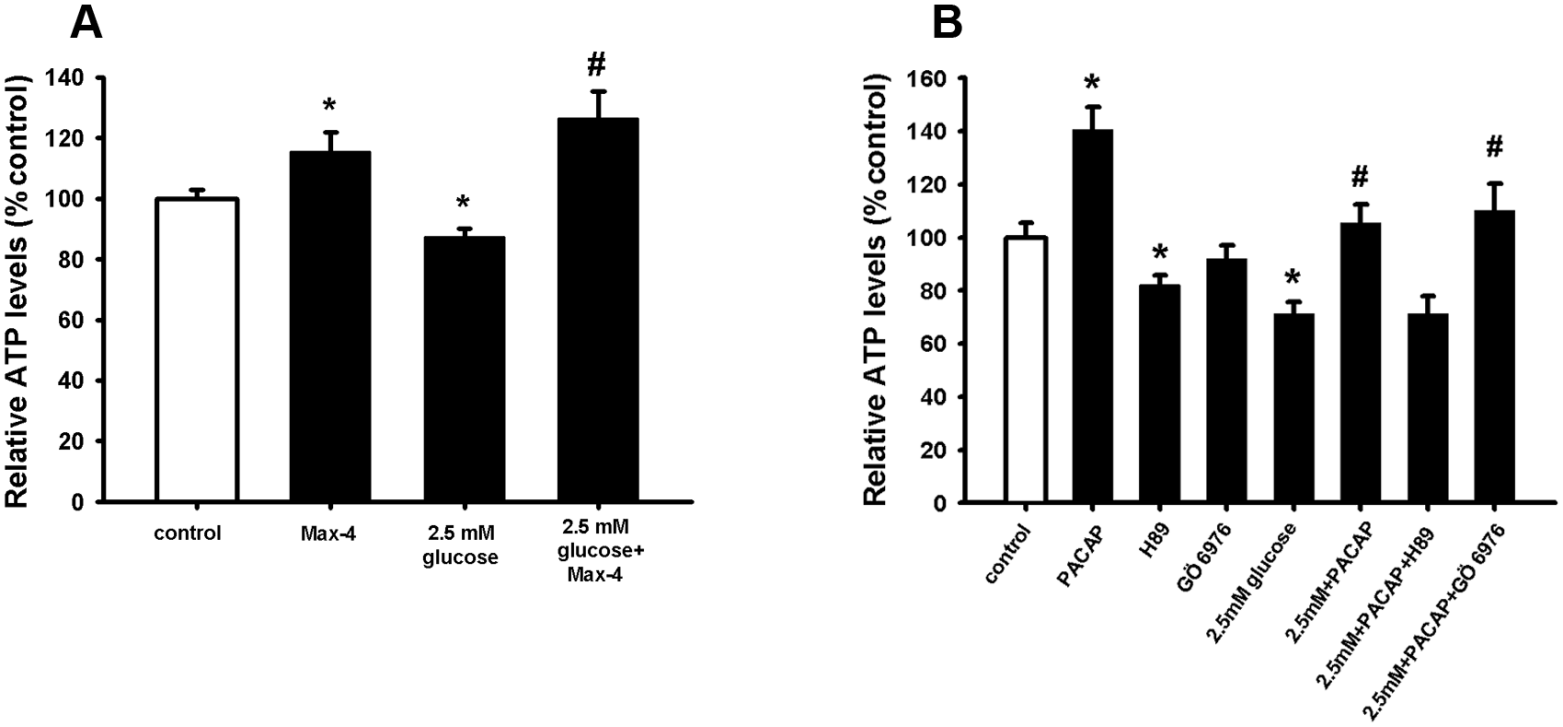
PAC-1 activation counteracts impaired NSCs under hypoglycaemic *milieu via* the PKA-dependent pathway. **(A, B)** NSCs were plated as single cells. Prior to 2.5mM glucose addition cells were incubated with PACAP (100 nM), Max-4 (30 nM), H89 (1 uM) and Gö6976 (1 uM) for 10 min. After 24 hours incubation, intracellular ATP levels were measured. Data are shown as mean ±SEM (A, n = 15–50; B, n = 16–28). Kruskal-Wallis followed by Dunn’s test was used. Differences were considered significant at P<0.05. * denotes P <0.05 compared with control, # denotes P<0.05 compared to 2.5mM glucose.

Previous works by others and us have shown that PACAP increases NSC viability through the activation of PAC-1 receptor [43, 47]. Therefore, to determine whether the protective effect of PACAP against hypoglycaemia was mediated by PAC-1 activation, NSCs were pre-treated with the specific agonist for PAC-1; Max-4 (30 nM) [61], before exposing the cells to low glucose for 24 hours. The results in Fig 4A show that Max-4 was able to counteract the negative hypoglycaemic effect on NSCs viability similarly to PACAP (see Fig 2C above). Moreover, we were able to challenge the NSCs with the PAC-1/VPAC2 antagonist PACAP 6–38 [62], where we saw PACAP protective effect was significantly diminished in the presence of the this antagonist and low glucose (data not shown).

PAC-1 has six intracellular splice variants dictating whether the PAC-1-mediated signalling pathway occurs through adenylate cyclase *via* protein kinase A (PKA) or through phospholipase Cγ (PLCγ) *via* PKC [63]. To determine whether the protective effect of PACAP against hypoglycaemia was mediated by PKA or PKC activation, two specific inhibitors were used: the PKC inhibitor Gö6976 and the PKA inhibitor H89. The results in Fig 4B show that the PACAP neuroprotective effect against hypoglycaemia was abolished in the presence of the PKA inhibitor H89, while it was unaffected in presence of the PKC inhibitor Gö6976. These results indicate that the adenylate cyclase/PKA signalling is responsible for the PAC-1-mediated neuroprotective effect against hypoglycaemia.

### Ex-4 did not mediate NSC protection under hypoglycaemic conditions

**Fig 5:**
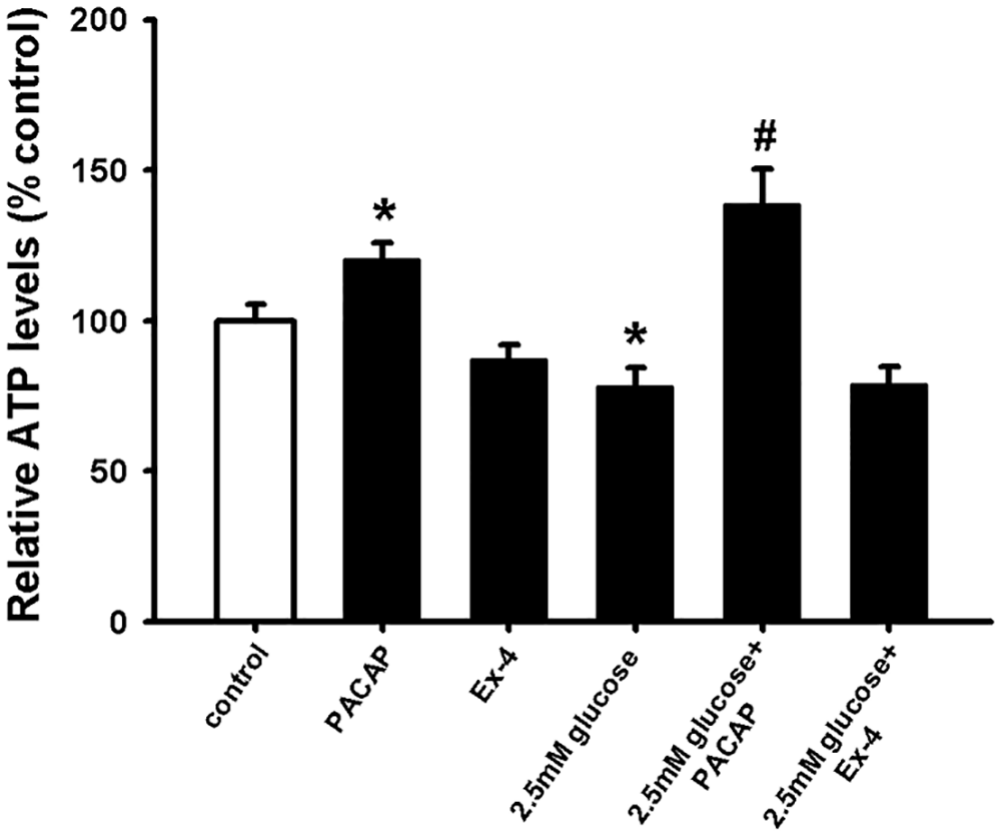
GLP-1R activation by Ex-4 did not protect NSCs from hypoglycaemic *milieu*. NSCs were plated as single cells. Prior to 2.5mM glucose addition cells were incubated with Ex-4 (10 nM) or PACAP (100 nM) for 10 min. After 24 hours incubation intracellular ATP levels were measured. Data are shown as mean ±SEM (n = 17–25). Kruskal-Wallis followed by Dunn’s test was used. Differences were considered significant at P<0.05. * denotes P <0.05 compared with control, # denotes P<0.05 compared to 2.5mM glucose.

Anti-diabetic therapies based on GLP-1R activation have been shown to be neuroprotective (see Intro) as well as to present very low risk for hypoglycaemia when combined with insulin therapy [64, 65]. To determine whether the GLP-1R agonist Ex-4 can counteract hypoglycaemia in NSC *in vitro*, we pre-treated NSCs with Ex-4 before adding low glucose for 24 hours. The results in Fig 5 show that Ex-4 could not counteract the decreased NSC viability induced by hypoglycaemia.

## Discussion

Hypoglycaemia is emerging as a highly pressing health concern as intensive glycaemic control has become the standard care in diabetes. Hypoglycaemic episodes are frequent following insulin treatment in T2D, in particular when insulin is given as a combination therapy with sulfonylureas [8, 9]. As a consequence, T2D patients can experience moderate or severe brain injury [20], including serious cognitive decline [4, 66].The deleterious effect of hypoglycaemia on neuronal survival are historically well known [67] and studies have shown that hypoglycaemia causes a cascade of biological effects that may cause ischemic cerebral damage in diabetic patients [9]. The putative consequences of hypoglycaemia, such as cognitive dysfunction, experienced by diabetic patients have been confirmed in experimental animal models of hypoglycaemia (without potential confounding factors) which have shown a strong association between learning and memory deficits and frequency of hypoglycaemia [28, 68]. Despite this, there is no effective therapy to treat hypoglycaemia-induced brain injury, neither there are, according to our knowledge, studies evaluating whether specific anti-hyperglycaemic drugs might act as neuroprotectants against hypoglycaemia-induced brain injury.

Adult neurogenesis is dynamically regulated in response to traumatic brain injuries and neurological diseases and disorders, suggesting that the regulation of this process can play an important role not only under physiological but also pathological conditions [69]. Whether and how adult neurogenesis is impacted by hypoglycaemia has been only investigated in a limited number of studies in hippocampus [18, 41, 42], in which the cellular and molecular mechanisms at the basis of this effect have not been extensively addressed. In one study, Suh *et al* showed increase of proliferation and neurogenesis in the rat hippocampus two weeks after inducing hypoglycaemia, yet a loss of progenitor cells after 4 weeks was demonstrated [18]. These data are in agreement with our results in SVZ-derived NSCs, showing that hypoglycaemia directly decreases NSC survival. A recent article from Ernst *et al*. [36] demonstrated that SVZ neurogenesis in the adult human brain is a crucial process to allow interneurons turnover in the striatum along the whole life. In addition, striatal stroke-induced neurogenesis from SVZ-derived NSCs has been proposed to play an important role in endogenous brain repair after stroke [70]. Based on these findings, we can speculate that hypoglycaemia-decreased NSC survival in the SVZ, under either normal or pathological conditions, could have detrimental effects on brain function and recovery after injuries such as stroke. If so, normalizing impaired adult neurogenesis in response to hypoglycaemia might provide a therapeutic strategy for treating T2D patients suffering from hypoglycaemia under glucose lowering treatments.

To identify potential drugs able to counteract the hypoglycaemia-mediated SVZ neurogenesis impairment and to characterize some of the mechanisms at the basis of this impairment, we have established an *in vitro* assay that mimics a hypoglycaemic *milieu* by exposing NSCs to low levels of glucose (≤ 2.5mM glucose). These glucose concentrations have been reported to be clinically relevant in hypoglycemic patients [71]. We show that hypoglycaemia induced by ≤ 2.5mM glucose impairs NSC viability in a dose-dependent manner and that this effect correlates with a modest increase in apoptosis (increased levels of cleaved-caspase 3). The data of cleaved-caspase 3 have been achieved by normalizing on β-actin which could be the reason why we did not reach statistical significance. A more precise normalization should have been performed on the total levels of caspase 3 and this is a limitation of the study.

Caspase activity also plays a critical role in ER stress-induced apoptosis. After the activation of the PERK pathway, increased expression of the transcription factor CHOP down-regulates Bcl-2 protein levels inducing activation of caspase-9 and caspase-3 resulting into apoptotic death [72, 73]. We show that hypoglycaemic conditions also activate ER stress in NSCs by increasing mRNA levels of CHOP. Altogether, these results indicate that hypoglycaemia increase apoptosis and ER stress. Although, Garcia de la Cadena *et al*. demonstrated activation of ER stress induced apoptosis by hypoglycaemia in hippocampal neurons *in vitro* [17], to our knowledge, our data are the first to show ER stress as potential mechanisms at the basis of the negative effects of hypoglycaemia on adult NSCs.

In the attempt to identify potential molecules able to counteract the neurogenesis impairment by hypoglycaemia, we identified PACAP as potent mediator of NSCs survival under hypoglycaemia. We selected PACAP based on three main reasons: 1) there is extensive literature about the neuroprotective effects mediated by this neuropeptide [(reviewed by Reglodi *et al* [74])] 2) it was recently shown that PACAP modulates neurogenesis *in vitro* and *in vivo* (see Intro) 3) PACAP has been shown to restore euglycaemia in response to acute hypoglycaemia by stimulating glucagon secretion [52].The surviving effect of PACAP against hypoglycaemia correlated with both counteracting hypoglycaemia-increased apoptosis and decreasing hypoglycaemia-induced ER stress. Furthermore, we showed that the protective effect of PACAP was specifically mediated *via* the PAC-1 receptor subtype through the adenylate cyclase/PKA signalling pathway. Although the neuroprotective effects mediated by PACAP are well known [74], the potential therapeutic effect of this peptide in response to hypoglycaemia has not been previously reported.

Several studies have shown that GLP-1 and GLP-1 analogues may delay cognitive decline in models of AD and Parkinson’s disease as well as to mediate neuroprotection in *in vitro* and *in vivo* models of diabetes and stroke [48, 51, 75]. Interestingly, GLP-1R agonists are associated with minimal risk of hypoglycaemia, since their metabolic effects are glucose-dependent [64, 65]. Although, the specific GLP-1R agonist Ex-4 has been reported to stimulate NSC viability and to counteract NSC apoptosis both *in vitro* and *in vivo* [48, 49], surprisingly, we could not achieve NSCs protection by Ex-4 in response to hypoglycaemia. It is hard to speculate why we could not detect any protective effect by Ex-4 in our *in vitro* system. However, due to the above mentioned neuroprotective properties of Ex-4 in normal or hyperglycaemic conditions, a plausible explanation could be that Ex-4 needs glucose to exert cellular protection.

In conclusion, we have established an *in vitro* system where to study the effects of hypoglycaemia on SVZ-derived primary adult NSCs. We show that hypoglycaemia decreases NSCs viability in correlation to increased ER stress and likely also apoptosis. Moreover, we report a strong neuroprotective effect of PACAP on NSCs exposed to a hypoglycaemic *milieu* that is mediated by PAC-1 activation *via* the adenylate cyclase/PKA signalling. These results motivate further *in vivo* studies with PAC-1 agonists aiming to identify efficacious compounds for the treatment of neuronal complications induced by hypoglycemia-induced NSC impairment.

## Supporting Information

S1 ARRIVE Checklist The ARRIVE guidelines checklist for reporting *in vivo* experiments in animal research. (PDF)

S2 ARRIVE Checklist The ARRIVE guidelines checklist for reporting *in vivo* experiments in animal research. (PDF)

S1 Data Individual data points presented on Figs 1–5. (XLSX)

S1 Fig Immunoblots (1–3) used for semiquantitative analyses of cleaved caspase-3 and Bcl-2. Abbreviations: Ctr = control, Hypo = 2.5mM glucose, Hypo+PACAP = 2.5mM glucose+PACAP, K+—positive control: NSCs treated with 0.3mM palmitate. Bands taken for analyses are framed in red.
(DOCX)
