# Supplementary material for: Pituitary Adenlylate Cyclase Activating Peptide Protects Adult Neural Stem Cells from a Hypoglycaemic milieu
Source: PLoS One. 2016 Jun 15;11(6):e0156867. doi: 10.1371/journal.pone.0156867 (PMC4909203; doi:10.1371/journal.pone.0156867)
Supplement: S1 ARRIVE Checklist — (PDF) [file pone.0156867.s001.pdf]

ARRIVE

The ARRIVE Guidelines Checklist

Animal Research: Reporting In Vivo Experiments

Carol Kilkenny[1], William J Browne[2], Innes C Cuthill[3], Michael Emerson[4] and Douglas G Altman[5]

[1]*The National Centre for the Replacement, Refinement and Reduction of Animals in Research, London, UK,* [2]*School of Veterinary Science, University of Bristol, Bristol, UK,* [3]*School of Biological Sciences, University of Bristol, Bristol, UK,* [4]*National Heart and Lung Institute, Imperial College London, UK,* [5]*Centre for Statistics in Medicine, University of Oxford, Oxford, UK.*

	ITEM	RECOMMENDATION	Section/ Paragraph
Title	1	Provide as accurate and concise a description of the content of the article as possible.	page: 1
Abstract	2	Provide an accurate summary of the background, research objectives, including details of the species or strain of animal used, key methods, principal findings and conclusions of the study.	page: 2
INTRODUCTION			
Background	3	a. Include sufficient scientific background (including relevant references to previous work) to understand the motivation and context for the study, and explain the experimental approach and rationale.	page: 3-5
		b. Explain how and why the animal species and model being used can address the scientific objectives and, where appropriate, the study's relevance to human biology.	page: 3-5
Objectives	4	Clearly describe the primary and any secondary objectives of the study, or specific hypotheses being tested.	page: 5
METHODS			
Ethical statement	5	Indicate the nature of the ethical review permissions, relevant licences (e.g. Animal [Scientific Procedures] Act 1986), and national or institutional guidelines for the care and use of animals, that cover the research.	page: 9
Study design	6	For each experiment, give brief details of the study design including:	
		a. The number of experimental and control groups.	page: 5-6
		b. Any steps taken to minimise the effects of subjective bias when allocating animals to treatment (e.g. randomisation procedure) and when assessing results (e.g. if done, describe who was blinded and when).	N/A in vitro experiment
		c. The experimental unit (e.g. a single animal, group or cage of animals).	
		A time-line diagram or flow chart can be useful to illustrate how complex study designs were carried out.	
Experimental procedures	7	For each experiment and each experimental group, including controls, provide precise details of all procedures carried out. For example:	N/A in vitro experiment
		a. How (e.g. drug formulation and dose, site and route of administration, anaesthesia and analgesia used [including monitoring], surgical procedure, method of euthanasia). Provide details of any specialist equipment used, including supplier(s).	N/A in vitro experiment
		b. When (e.g. time of day).	
		c. Where (e.g. home cage, laboratory, water maze).	— " —
		d. Why (e.g. rationale for choice of specific anaesthetic, route of administration, drug dose used).	page: 9
Experimental animals	8	a. Provide details of the animals used, including species, strain, sex, developmental stage (e.g. mean or median age plus age range) and weight (e.g. mean or median weight plus weight range).	page: 5
		b. Provide further relevant information such as the source of animals, international strain nomenclature, genetic modification status (e.g. knock-out or transgenic), genotype, health/immune status, drug or test naïve, previous procedures, etc.	N/A in vitro experiments
