# Supplementary figures and images for: Pituitary Adenlylate Cyclase Activating Peptide Protects Adult Neural Stem Cells from a Hypoglycaemic milieu
Source: PLoS One. 2016 Jun 15;11(6):e0156867. doi: 10.1371/journal.pone.0156867 (PMC4909203; doi:10.1371/journal.pone.0156867)

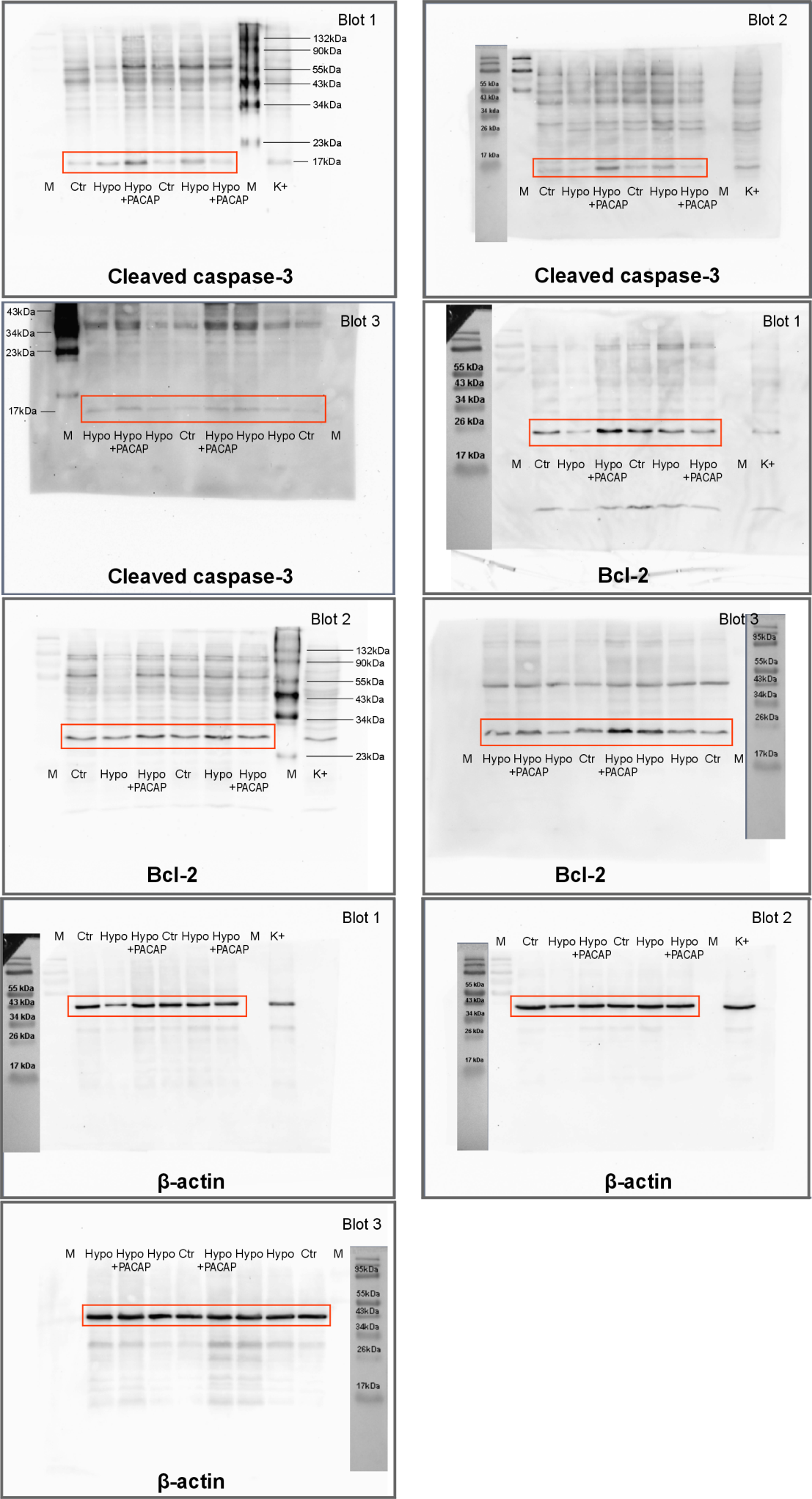

Supplement: S1 Fig — Abbreviations: Ctr = control, Hypo = 2.5mM glucose, Hypo+PACAP = 2.5mM glucose+PACAP, K+—positive control: NSCs treated with 0.3mM palmitate. Bands taken for analyses are framed in red. (DOCX) [file pone.0156867.s004.docx]
